# The Effect of Interventions Based on the Information-Motivation-Behavioral Skills Model on the Human Papillomavirus Vaccination Rate Among 11-13-Year-Old Girls in Central and Western China: Protocol for a Randomized Controlled Trial

**DOI:** 10.2196/58873

**Published:** 2024-11-19

**Authors:** Shu Jing, Yijin Wu, Zhenwei Dai, Shenglan Tang, Xiaoyou Su, Youlin Qiao

**Affiliations:** 1 School of Population Medicine and Public Health Chinese Academy of Medical Sciences & Peking Union Medical College Beijing China; 2 National Health Commission Key Laboratory of Mental Health Peking University Institute of Mental Health Peking University Beijing China; 3 National Clinical Research Center for Mental Disorders Peking University Sixth Hospital Peking University Beijing China; 4 Duke Global Health Institute Duke University Durham, NC United States; 5 Global Health Research Center Duke Kunshan University Kunshan China; 6 Department of Cancer Epidemiology National Cancer Center/National Clinical Research Center for Cancer/Cancer Hospital Chinese Academy of Medical Sciences and Peking Union Medical College Beijing China

**Keywords:** human papillomavirus, HPV, HPV vaccine, vaccine hesitancy, information-motivation-behavioral skills model, HPV vaccination rate, randomized controlled trial, vaccination rate, China

## Abstract

**Background:**

Persistent infection of high-risk human papillomavirus (HPV) can lead to cervical intraepithelial neoplasia, cervical cancer, and even death. HPV vaccination for girls aged 9-14 years can effectively prevent the occurrence of cervical cancer. Some Chinese provinces and cities have launched free HPV vaccination programs for school-age girls; however, due to the lack of supportive government policies, the high price and supply shortage of HPV vaccines, and vaccine hesitancy, some parents refuse to vaccinate their daughters.

**Objective:**

This protocol reports the design of a randomized controlled trial (RCT) aiming to explore the efficacy of a digital HPV vaccination education intervention based on the information-motivation-behavioral skills (IMB) model in improving the HPV vaccination rate among 11-13-year-old girls in central and western China.

**Methods:**

A multicenter intervention study based on an online applet will be conducted in December 2024, and about 750 eligible parents of 11-13-year-old girls will be assigned in a 1:1 ratio to an intervention group receiving 7-day digital HPV vaccination education based on the IMB model or a control group using non-HPV publicity materials. Free HPV vaccination pilot projects will be carried out among this population by our research team in central and western China (some parents might refuse to vaccinate their daughters). All participants will be asked to complete online questionnaires at baseline; postintervention; and 1 week, 1 month, and 3 months after the intervention.

**Results:**

The primary outcome of this study will be receipt of the first HPV vaccination within 3 months. Data will be analyzed based on an intention-to-treat approach, and Stata 16.0 will be used for statistical analysis.

**Conclusions:**

This study aims to improve the HPV vaccination rate among 11-13-year-old girls and will examine the impact of a digital HPV vaccination education intervention based on the IMB model. The findings of this study may offer promising intervention measures for HPV vaccine hesitancy in low-health-resource areas in the future.

**Trial Registration:**

Chinese Clinical Trial Registry, ChiCTR2300067402; https://tinyurl.com/v5zt4hc9

**International Registered Report Identifier (IRRID):**

PRR1-10.2196/58873

## Introduction

Human papillomavirus (HPV) is one of the most common sexually transmitted infections that can cause various genital diseases among women [1]. Persistent high-risk HPV infection is closely associated with the occurrence of cervical and other cancers [2]. According to the worldwide cancer statistics released by the International Agency for Research on Cancer, there were 604,000 new cases of cervical cancer and 342,000 deaths due to cervical cancer in 2020 worldwide [3]. The incidence of cervical cancer ranked third among malignant tumors, and the estimated death toll ranked second among deaths due to malignant tumors in Chinese women aged 15-44 years in 2020 [3]. In the past decade, the incidence and mortality of cervical cancer in China have shown an upward trend year on year [4]. Overall high-risk HPV infection rates in mainland Chinese women are about 19.0%, with subtypes 16, 52, 58, 53, and 18 being the most common infections [5].

Cervical cancer is considered near completely preventable through the primary and secondary prevention measures of HPV vaccination and screening [6]. In November 2020, the World Health Organization (WHO) released the *Global Strategy to Accelerate the Elimination of Cervical Cancer as a Public Health Problem* [4]. The WHO position paper recommended that young adolescent girls aged 9-14 years be the primary target group for HPV vaccination [7]. Relevant clinical trials and cohort studies have verified the safety and efficacy of HPV vaccines among this population [8]. In some high-income countries, the incidence of cervical cancer apparently decreased 10-12 years after widespread HPV vaccination [9,10]. However, effective prevention strategies were not applied adequately, with only 15% of girls of the target age vaccinated with the full course of HPV vaccines in 2019 [11]. In China, the HPV vaccination rate among girls aged 9-14 years was less than 1% in 2020, which is lower than the current world average of 20% [12,13]. As a result, improving the HPV vaccination rate among girls is an urgent need in China.

Chinese authorities approved HPV vaccines in 2016 [14]. Since 2020, some provinces and cities in China, especially those with good financial status, such as Guangdong Province and Fujian City, have launched free HPV vaccination programs for school-age girls [15]. However, without supportive government policies, the high price and supply shortage of HPV vaccines have impacted accessibility and affordability in the central and western regions of China [16,17]. In addition to the financial considerations and accessibility issues, vaccine hesitancy is also a major barrier to vaccine coverage [18]. Vaccine hesitancy is defined as the refusal or delay of vaccination when immunization services are available, which means low vaccine acceptance despite the availability of vaccination services [16]. Given the young age of the HPV vaccine target population, this study will focus on HPV vaccine hesitancy among the parents of girls aged 9-14 years. A recent study in mainland China reported that 53.9% of the guardians of secondary school girls aged 12-19 years were HPV vaccine hesitant, mainly because of a lack of knowledge of HPV vaccines and insufficient communication from reliable sources of information [19]. Previous studies have demonstrated that health interventions targeting parents can effectively improve vaccine coverage among girls. In 2018, an intervention using health education manuals and reminder letters was implemented among parents of 11-12-year-old girls in New York State, which increased the HPV vaccination rate from 24.1% to 75% [20]. Research on East African immigrant mothers conducted 10 HPV vaccine–related health education forums to increase HPV vaccine–related knowledge, improve attitude, and increase the intention to vaccinate adolescent children. Results from this study showed that mothers who attended the forums showed a more positive attitude and an intention to getting their daughters vaccinated [21]. Therefore, vaccine hesitancy has been shown to be a major obstacle to HPV vaccination among targeted girls.

**Figure 1:**
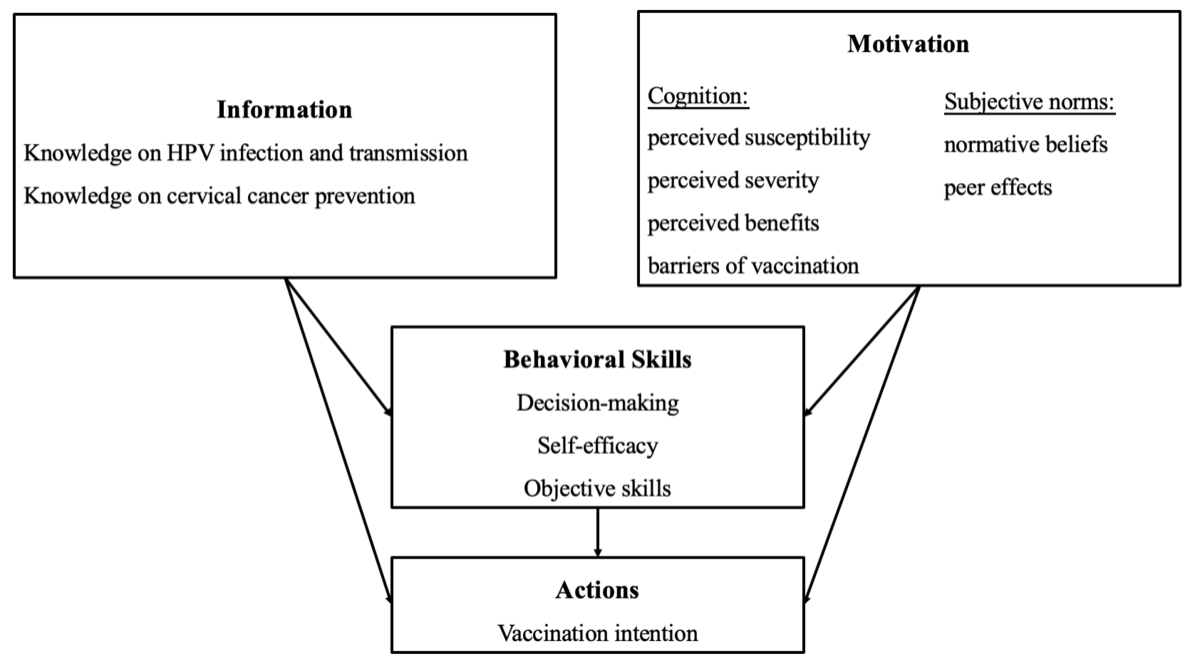
Framework of the IMB model used in the study. HPV: human papillomavirus; IMB: information-motivation-behavioral skills.

In 2023, our research team initiated a free HPV vaccination pilot project among low-health-resource areas in China, which may last for at least 2 years, aiming to provide girls aged 11-13 years with HPV vaccines. Considering the possible HPV vaccine hesitancy among parents, this study will use an intervention for parents to improve their vaccination intention and increase the vaccination rate among their daughters, guided by the information-motivation-behavioral skills (IMB) model. The IMB model is a validated approach to predicting and promoting health behavior performance by providing relevant information, personal and social motivation, and behavioral skills [22,23]. It has been widely used in behavior interventions over the past decade among different populations, such as HIV prevention among Indian truck drivers, improvement of maternal breastfeeding efficiency in pregnant women with hepatitis B virus (HBV) infection, and antiretroviral therapy (ART) adherence improvement among HIV-positive patients in southwestern China [24-26]. In terms of HPV vaccine interventions in China, the IMB model has been proven to effectively increase the willingness of HPV vaccination, when combined with an online program [27]. This study will use the IMB model, which asserts that information related to HPV infection and cervical cancer prevention, HPV vaccination motivation, and behavioral skills are fundamental determinants of HPV vaccination (Figure 1) [28].

We hypothesize that a digital HPV vaccination education intervention based on the IMB model will motivate parents to vaccinate their children against HPV, thus significantly improving the local HPV vaccination rate among 11-13-year-old girls (who are in the senior grade of primary school). Therefore, the primary objective of this study is to improve the HPV vaccination rate among 11-13-year-old girls in central and western China. Through this study, we aim to explore the potential influencing factors and mechanisms of parents’ hesitancy to vaccinate their daughters against HPV. In addition, we will discuss the intervention effects of 7-day digital HPV vaccination education based on the IMB model on the parents. The ultimate goal is to evaluate the efficacy of such intervention measures against HPV vaccine hesitancy in order to help improve the HPV vaccination rate among 11-13-year-old girls in China in the future.

## Methods

### Trial Design

**Figure 2:**
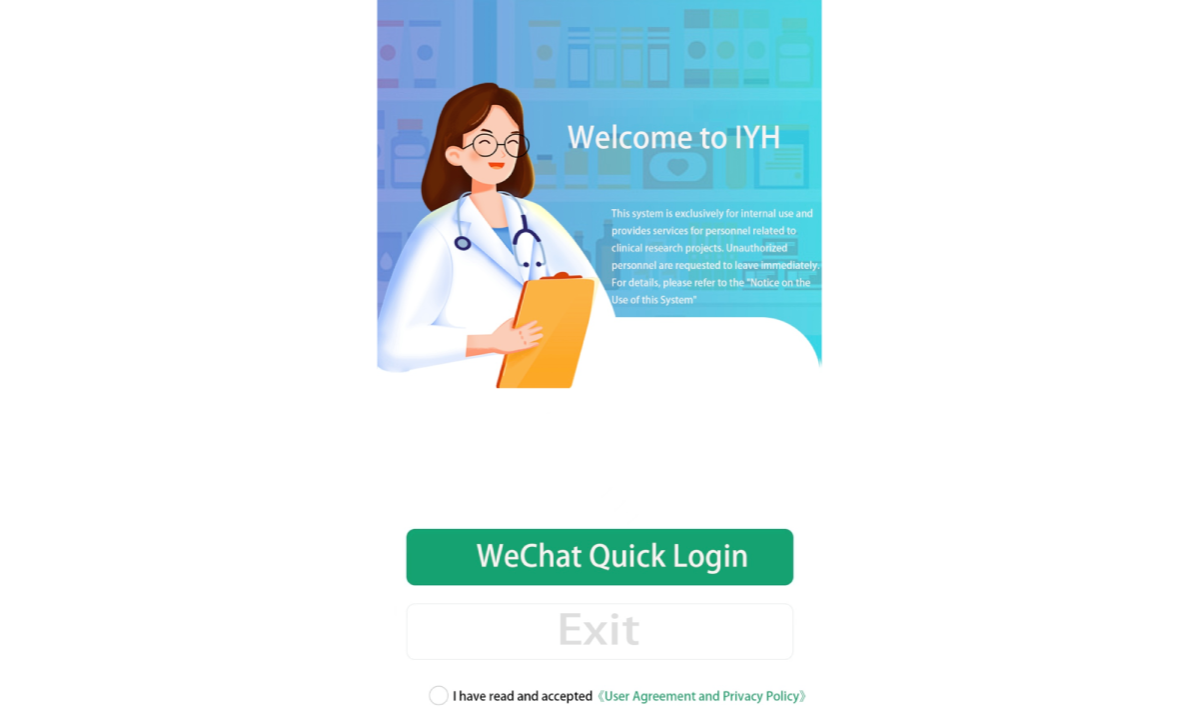
Interface of the "Yi Yan Health" mini applet. IYH: Yi Yan Health mini-program.

**Figure 3:**
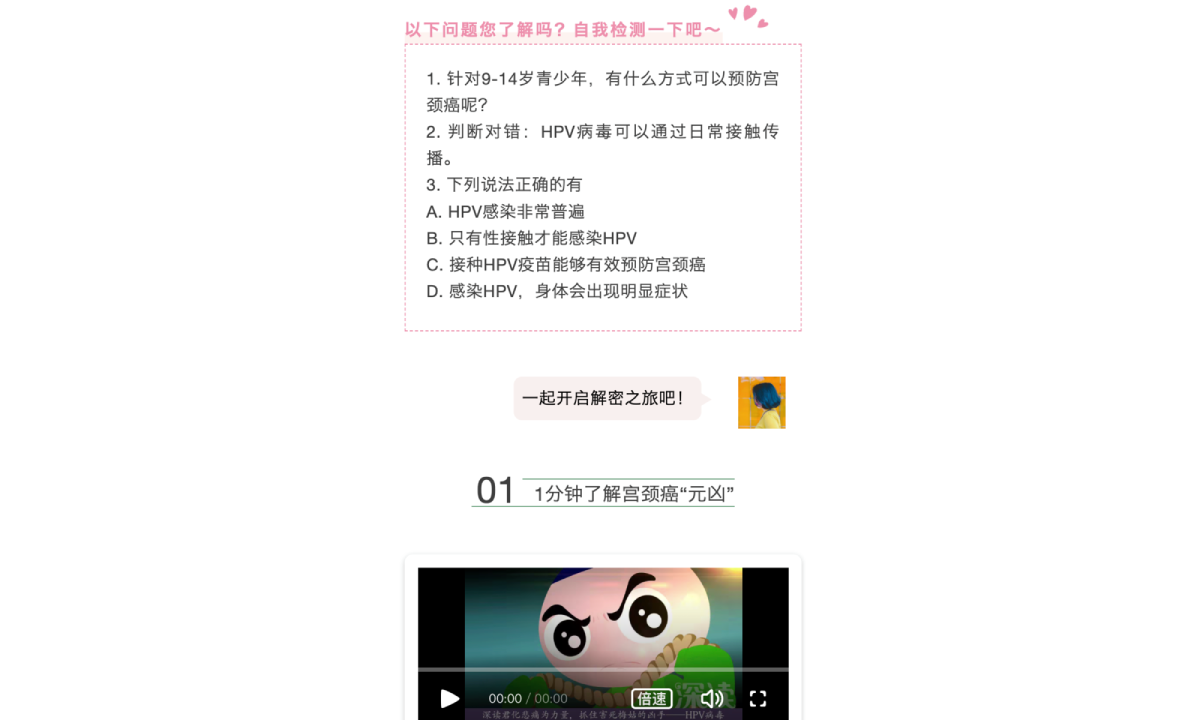
Mobile app intervention material sample.

This will be a double-blind randomized controlled trial (RCT) of an intervention based on an online applet. Before the intervention, the research team will carry out free HPV vaccination pilot projects for 11-13-year-old girls in 4-5 study sites in low-health-resource areas in China. After the first stage of the HPV vaccination program is completed, 11-13-year-old girls’ parents who choose not to vaccinate their daughters will be invited to our intervention study, and those who are willing to participate will be guided to register and log on to the “Yi Yan Health” mini applet (Figure 2), which was independently developed by our research team and can be used for mobile app–based education and questionnaire collection. Next, all participants will be randomly divided into 2 groups: (1) an intervention group, which will receive a baseline questionnaire, 7-day digital HPV vaccination education based on the IMB model (with a sample shown in Figure 3), and follow-up right after the intervention period and 1 week, 1 month, and 3 months after the intervention, and (2) a control group, which will only receive popular science education that is not related to HPV vaccines; these non-HPV publicity materials will be uploaded at the same time every day as the intervention group to maintain parents’ interest in the control group. Meanwhile, participants in the control group will also need to complete questionnaires simultaneously with the intervention group. After the intervention trial, we will provide the control group with the same IMB-based HPV vaccine intervention materials free of charge for ethical considerations. Our free HPV vaccination pilot program will also continue until at least half a year after the intervention to ensure that all 11-13-year-old girls whose parents are willing to vaccinate their daughters can receive the first HPV vaccination during or after the intervention.

### Settings

Our research team will conveniently select 5 cities or counties in central and western China as research sites. Local hospitals or medical schools will provide assistance in the recruitment of participants and other implementation issues of the project. All researchers in this study were required to have a professional background in medicine or public health and received unified training conducted by the research team before the start of the study. This study is scheduled to start in September 2024 and is expected to last for about 12 months. This study was registered on the Chinese Clinical Trial Registry (ChiCTR 2300067402, registration date January 6, 2023).

### Ethical Considerations

The subjects of this study are 11-13-year-old girls’ parents who do not choose to get their daughters vaccinated against HPV. Due to the fact that HPV vaccination for children in China requires parental informed consent, we will only recruit parents instead of their daughters in this study.

In adherence to normative documents, including the Declaration of Helsinki (revised by the World Medical Association in October 2013) and the “Approach to the Ethical Review of Biomedical Research Involving Humans” issued by the National Health Commission of the People’s Republic of China, Research Ethics Committee, Chinese Academy of Medical Sciences & Peking Union Medical College, approved this research (ID: CAMS&PUMC-IEC-2022-077) on November 30, 2022, following thorough review and discussion. Informed consent will be obtained from all participants, with a clear explanation of the project, including confidentiality agreements and conditions for opting out at any time.

If parents accept the free HPV vaccination for their daughter, they will need to pay a Chinese yuan (CNY) 20 (~US $3) injection fee to the local health facility, which is the same as the injection fee for other vaccines. Participants will incur no additional costs by participating in this study. Additionally, they will benefit from learning about HPV, HPV vaccines, and cervical cancer prevention. Participants can choose to vaccinate their daughters with free HPV vaccines offered by the government or decline. This project aims to improve HPV vaccine coverage among girls aged 11-13 years in low-health-resource areas in China, provide valuable insights into promoting HPV vaccination nationwide, and, ultimately, contribute to reducing and potentially eliminating cervical cancer in China.

### Patient and Public Involvement

After project introductions by the researchers, those who are interested in this study will be recruited. Once participants sign the informed consent form, the researchers will complete eligibility checklists, and those who fail to meet the inclusion criteria will be removed. All information will be stored on an encrypted laptop. Participants will also have the right to freely obtain more information at any time and will be allowed to freely withdraw from the study without restrictions at any research stage.

### Sample Size

According to the purpose of intervention effect evaluation, the comparison formula of 2 independent rates is used to calculate the required sample size. To date, there are no studies on the effects of a mobile app–based intervention for 11-13-year- old girls in central and western China to improve the HPV vaccination rate. For this reason, the anticipated effect size is largely based on findings of similar studies derived from the relevant literature in other countries. In this study, according to the literature review and the results of our research team’s free HPV vaccination campaign conducted in Ordos City, Inner Mongolia Autonomous Region, China, in 2020, about 30% of parents in the control group refused to vaccinate their daughter (Pc), while parents in the intervention group who refused are expected to be 20% lower than those in the control group (Pt): α=.05 (2-sided), β=.2 [29-31]. Considering the possible dropout (20%), the intervention and control groups need 74 effective samples each, totaling 148, and each project site needs to recruit about 150 participants. There will be 750 participants in 5 project sites, 375 (50%) in the intervention group and 375 (50%) in the control group.

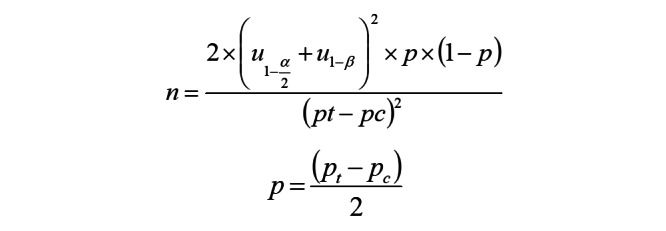



### Eligibility Criteria

The inclusion criteria are as follows:

- Parents who have refused to vaccinate their 11-13-year-old daughters against HPV after being offered the first dose of vaccination
- Parents who have the local household registration of the research site
- Parents who have an electronic device with internet access, such as a smartphone, a tablet, or a laptop
- Parents who are able to read and understand the questionnaire
- Parents who understand the vaccination procedure and volunteer to participate in this study

The exclusion criteria are as follows:

- Parents whose daughters have contraindications for HPV vaccination or other physical conditions that are not suitable for HPV vaccination
- Parents who have participated in similar research in the past 6 months

### Recruitment

In 5 regions with free HPV vaccination pilot projects, parents who refuse to vaccinate their daughters and who meet the inclusion criteria will be enrolled into this study. After providing informed consent, all participants will register and log on to the mini applet, and they will then be randomly assigned to the intervention group or the control group in a 1:1 ratio using the randomization algorithm embedded in the mini applet.

### Intervention

#### Intervention Group

All participants in the intervention group will need to log on to the mini applet through a computer or a smart phone and will receive 7-day digital HPV vaccination education based on the IMB model. Our research team has developed intervention materials after reviewing the relevant literature and conducting multiple rounds of panel discussions with a group of experts, including 1 cancer epidemiologist, 1 infectious disease epidemiologist, 1 researcher from the project center, and 1 senior manager from a media company. This 7-day digital HPV vaccination education intervention was previously applied by our research team in a project on HPV vaccination willingness of Chinese female college students and achieved positive results [14,32]. However, since digital education aims to target different participants in this study, the researchers have worked with the expert panel to adjust and optimize the previous content to better meet the needs of the parents of 11-13-year-old girls before the start of intervention.

According to the IMB model, digital HPV vaccination education is divided into 3 modules and 6 themes, with each module containing 2 themes. The first module is information, which is the initial prerequisite for promoting healthy behaviors. Module 1 has 2 themes: (1) HPV infection and related diseases and (2) high-risk factors of HPV infection and how to prevent HPV infection. The second module is motivation, which mainly refers to personal attitudes toward health behavior and related social motivation. Personal attitudes include the understanding of HPV susceptibility, the expectation of costs and benefits of taking preventive actions, and the experience and willingness to take actions; social motivation includes social support and social values. Module 2 has 2 themes: (3) introduce the benefits of HPV vaccines so as to encourage parents to vaccinate their daughters and (4) describe the harm and susceptibility of HPV infection through a true story to motivate parents to take action to prevent their daughters from contracting HPV infection. The third module is behavioral skills, which mainly emphasizes the improvement of personal objective skills and self-efficacy. Module 3 has 2 themes: (5) how to use personal objective skills to make firm decisions and communicate effectively with family members to obtain understanding and support for daughters’ HPV vaccination and (6) the concept and function of self-efficacy and ways to improve self-efficacy and promote the transformation of motivation into behaviors.

Each of the 6 thematic courses will be uploaded to the mini applet and will take participants about 10-15 minutes to read and learn. In addition, the mobile app–based HPV vaccination education contains a high level of interactivity with the following learning forms: reading text, viewing pictures, small videos pictures watching, and taking quizzes. The intervention will last for 1 week, and data will be collected at baseline ( 1 week before the intervention); postintervention; and 1 week, 1 month, and 3 months after the intervention. All the data will be obtained for statistical analysis.

#### Control Group

The control group will receive health promotion materials unrelated to HPV vaccines, and the researchers will distribute the questionnaire to both intervention and control groups simultaneously through the same mini applet. After 3 months of follow-up, participants in the control group will receive the same health education about HPV vaccines as the intervention group, free of charge for ethical considerations.

### Adherence

The intervention will be stopped at the participants’ request if they find anything harmful about it, and participants have the right to refuse to answer some sensitive questions in the questionnaire or to withdraw from the study at any time.

Before the intervention, the researchers will introduce the importance and expected benefits of this study. During the intervention, once the course is uploaded, the mini applet will automatically send a text message to participants every day to remind them to learn until they have completed the course. The researchers can also supervise participants by their learning duration, which will be automatically recorded by the backend database of the mini applet. During follow-up, similarly, the mini applet will automatically send a text message to participants every day before they complete the questionnaire, and the number of completed questionnaires will be used to evaluate the adherence of participants to the entire trial process. Finally, we will express our gratitude to all participants for their involvement and encourage them to complete the study through nonmaterial incentives, such as by answering their questions related to HPV or cervical cancer during the research.

### Measures

#### Outcome Measurements

The questionnaire in this study is divided into 4 sections: general demographic data, current HPV vaccination status, parents’ hesitancy and attitudes toward HPV vaccines, and potential factors related to HPV vaccine hesitancy. The questionnaire in the study is designed in Chinese to ensure that participants can understand and respond to the questions.

#### Primary Outcome: HPV Vaccination Status

The ultimate goal of this study is to improve the HPV vaccination rate among 11-13-year-old girls. This indicator will be measured by the proportion of parents who choose to vaccinate their daughters against HPV within 3 months after the intervention. To ensure the authenticity of HPV vaccination, the vaccination status will be checked by the registration history in the information systems of local health agencies, which have already collaborated with us, rather than being self-reported by participants. The HPV vaccination behaviors will be strictly verified by our researchers, and those who are just “willing” to be vaccinated will not be included in the positive results.

#### Secondary Outcomes: Parents’ Hesitancy and Attitudes Toward HPV Vaccines

We will measure parents’ hesitancy and attitudes toward HPV vaccines in 3 parts. The first part is about general vaccination attitudes, which will be used to investigate participants’ attitudes toward other common vaccines recommended by the Chinese government. The second part is about HPV vaccine hesitancy, which will be used to assess the degree of HPV vaccine hesitancy among parents. The third part is about vaccine confidence used, which will be to investigate participants’ confidence in HPV vaccines.

#### Other Outcomes: Potential Factors Related to HPV Vaccine Hesitancy

To explore the potential factors associated with HPV vaccine hesitancy, our research team has designed a section based on the IMB theoretical framework to assess parents’ HPV-related information, vaccination motivation, and behavioral skills to further explore the factors associated with HPV vaccine hesitancy. We will also directly ask participants what factors can affect their daughters’ HPV vaccination, thereby investigating the reasons for their vaccine hesitancy.

### Blinding

Although participants will not be blinded because of the nature of the intervention, blinded evaluation will be adopted in our study. In this study, all researchers involved in outcome assessments will be blinded to the group allocation. During the intervention, only unblinded research assistants in each center will be aware of the allocation and will be in contact with participants. In addition, all data analysis will be completed by personnel who are blinded to the intervention assignment. Participants will be unblinded after the follow-up survey.

### Data Collection, Management, and Monitoring

The questionnaire will be distributed via the mini applet, and all completed questionnaires will be automatically saved into the read-only web-based database to ensure authenticity of the data. Moreover, our researchers will promptly check the collected data after each questionnaire survey. Regarding integrity of the data, all questions will be self-administered and set as mandatory to ensure that all submitted questionnaires have no missing data.

### Statistical Analysis

Stata 16.0 will be used for statistical analysis. The distribution of categorical variables will be described by frequencies and percentages, and continuous variables conforming to normal distribution will be described by means (SDs). Chi-square tests will be performed to compare the differences between different groups. The *t* test or 1-way ANOVA will be performed to compare the mean scores between groups on the same continuous and dependent variables. Logistic regression will be conducted to analyze the factors associated with HPV vaccine hesitancy. Repeated measurement data at baseline; postintervention; and 1 week, 1 month, and 3 months after the intervention will be analyzed using generalized estimation equations (GEEs). Significant variables will be included in baseline chi-square analysis, and we will also assess group (intervention and control), time (baseline; immediately after the intervention; and 1 week, 1 month, and 3 months after the intervention), and time×group interactions, with the interaction indicating a differential change by group from baseline to the end of the trial. Statistical significance will be set at *P*<.05 (2-sided).

**Results**

This study protocol is reported following SPIRIT (Standard Protocol Items: Recommendations for Interventional Trials) reporting guidelines. Table S1 in Multimedia Appendix 1 shows the SPIRIT diagram for the schedule of enrollment, intervention, and assessment.

**Figure 4:**
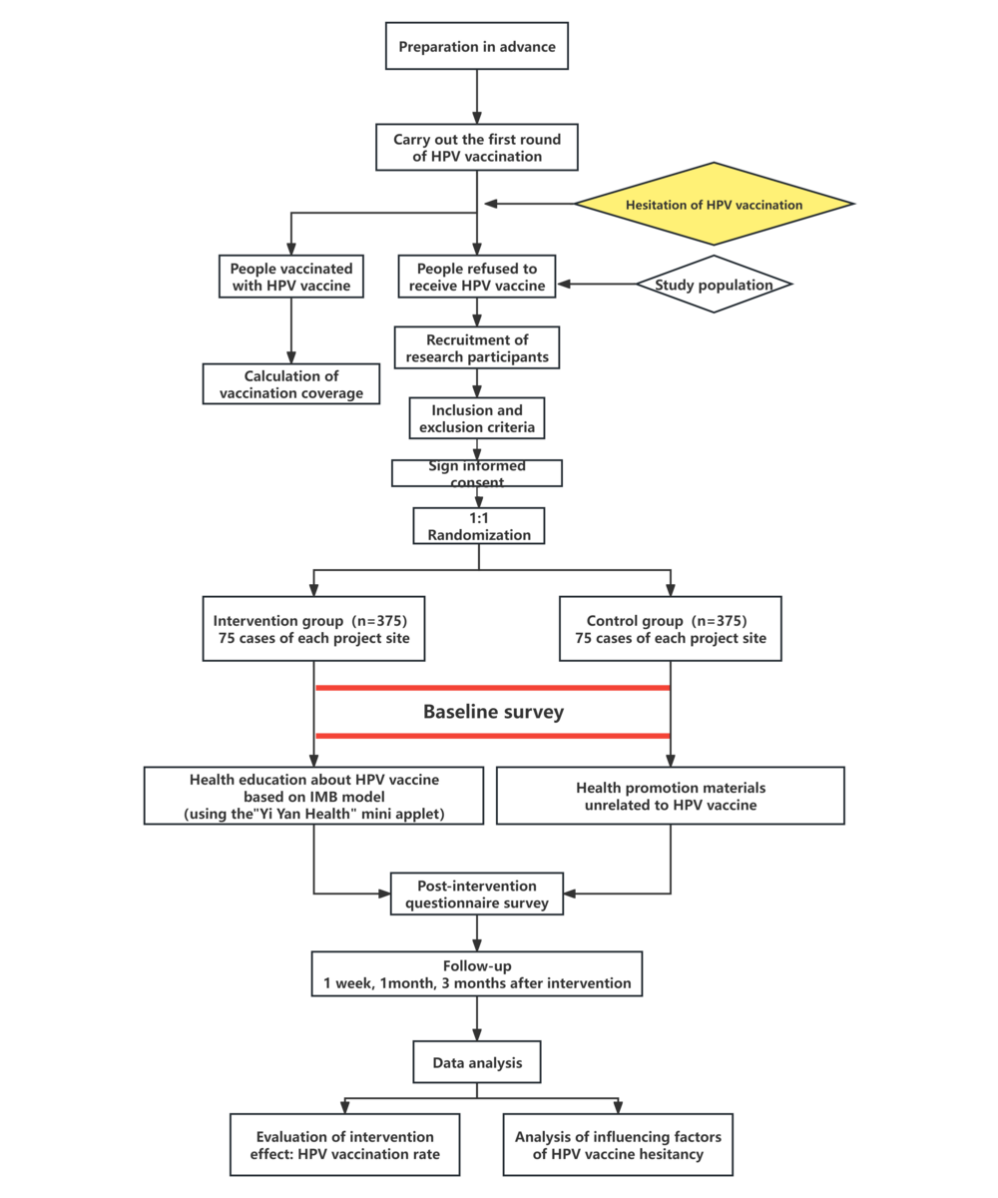
Flowchart of the protocol. HPV: human papillomavirus.

Figure 4 shows the flowchart of the protocol. See Table 1 for the structure design of the questionnaire and Table 2 for the structure design of the 7-day mobile app–based education intervention on HPV vaccines.

**Table 1:** Structure design of the 7-day digital education intervention on HPV^a^ vaccines.

Day	Content
1	Information about HPV infection
2	Information about HPV vaccines
3	Cherishing your health, actively preventing and controlling cervical cancer
4	Cervical cancer, a threat to women’s health
5	Introduction to effective decision-making and communication skills
6	Small tips to action promotion
7	Invitation to HPV vaccination

^a^HPV: human papillomavirus.

**Table 2:** Structure design of the questionnaire.

Section	Content or part
General social demographic data survey	Age, nationality, religious belief, permanent residence, parents’ education level, family monthly income (CNY^a^/month), whether any of the relatives has cancer, etc
HPV^b^ vaccination status	Whether parents chose to vaccinate their daughters against HPV within 3 months after the intervention or whether they took an appointment for HPV vaccination within 3 months
Vaccine hesitancy survey	General vaccination attitude survey HPV vaccine hesitancy survey Vaccine confidence survey
Factors resulting in HPV vaccine hesitancy	Parents’ HPV-related information, vaccination motivation, and behavioral skills (based on the IMB^c^ model) What factors can affect their daughters’ HPV vaccination?

^a^CNY: Chinese yuan.

^b^HPV: human papillomavirus.

^c^IMB: information-motivation-behavioral skills.

At the time of manuscript submission in October 2024, study recruitment was pending and was expected to start in November 2024. The expected results will be analyzed and prepared for publication in 2025.

## Discussion

### Summary

This multicenter RCT based on an online mini applet aims to reduce vaccine hesitancy among parents of 11-13-year-old girls in central and western China by using digital education measures based on the IMB model. By reducing vaccine hesitancy, the intervention will encourage parents to vaccinate their daughters against HPV, improve the coverage of HPV vaccines in China, and promote the elimination of cervical cancer in China. To the best of our knowledge, this study will be the first to evaluate whether the mobile app–based education intervention designed according to the IMB model can improve the HPV vaccination rate among 11-13-year-old girls in central and western China. In addition, this study will also explore the factors associated with parents’ HPV vaccine hesitancy, which may provide a much clearer reference for formulating an HPV vaccination policy and subsequent HPV vaccination rate intervention studies in China.

Our study will focus on girls in central and western China because the distribution of health resources in China is still not completely balanced, and the central and western regions have relatively fewer resources than the east [33,34]. Due to relatively scarce health resources, 11-13-year-old girls in central and western China are less knowledgeable about HPV and HPV vaccines [35]. In addition, compared to some eastern provinces of China, such as Guangdong, Fujian, and Shandong Provinces, which have launched free HPV vaccination campaigns for 11-13-year-old girls, it is much more difficult for girls in central and western China to obtain HPV vaccines. Therefore, it is more urgent and significant to carry out vaccination campaigns and intervention studies in central and western China to realize the target of eliminating cervical cancer in the country.

Previous studies have revealed that HPV vaccination can significantly reduce the risk of cervical cancer in women aged 9-45 years, and the earlier the vaccination, the better the preventative effect obtained [10]. At present, Finland, the United Kingdom, and other countries have implemented HPV vaccination plans for 11-13-year-old girls, and their current vaccination rates are high [36-38]. For example, more than 80% of 13-year-old girls in England have completed 2 doses of HPV vaccination, and similar high statistics are reported in other countries [38]. However, in China, HPV vaccines have been on the market for only a few years, and some parents remain skeptical or even hesitant of the vaccine, leading to an unsatisfactory coverage rate, especially among 11-13-year-old girls who are still studying in primary school [39]. A cross-sectional study conducted in Weihai, Shandong Province, China, about HPV vaccination willingness of parents of girls aged 9-17 years showed that only 19.32% of parents were willing to vaccinate their girls against HPV. These results suggest that Chinese parents have a high degree of HPV vaccine hesitancy and that relevant action is needed to change this attitude [40]. Additionally, an RCT conducted in the United States had parents of adolescents aged 11-17 years watch HPV vaccine health education videos in outpatient clinics, and 2 weeks later, the HPV vaccination rate among adolescents whose parents watched the videos was 3 times higher than among those whose parents did not watch the videos [41], which shows that health interventions targeting parents could effectively improve vaccine coverage among children and teenagers. Based on the aforementioned domestic and foreign evidence, it is necessary to explore effective strategies against HPV vaccine hesitancy among parents of 11-13-year-old girls in central and western China.

Evidence suggests that a change in behavior is the result of knowledge, psychology, and social factors, which indicates that the design of health education activities could be based on appropriate theories of behavior change [42,43]. The health intervention materials in this study are based on the IMB model, which improves the logicality and scientific integrity of the intervention. Our research team had previously used similar health education materials in an intervention for HPV vaccination willingness among female college students in mainland China, and the results revealed that the proportion of participants willing to receive HPV vaccines increased from 52.5% before the intervention to 59% after the intervention, while the proportion in the control group did not change [44]. These results indicate that the 7-day digital HPV vaccine education interventions based on the IMB model can improve the willingness of participants to receive HPV vaccines, which proves the effectiveness of mobile app–based education in female college students. Such effectiveness may be extended to other groups, such as parents of 11-13-year-old girls.

To the best of our knowledge, there is no research using the theory-based digital education method to improve the HPV vaccination rate among 11-13-year-old girls in central and western China. Regarding the study design, this double-blind RCT with the actual vaccine coverage rate as the main outcome may provide more innovative suggestions for the promotion of HPV vaccines. At present, because of the efforts of the Chinese government in infrastructure construction, mobile devices have become an integral part of daily life among the majority of Chinese people, including in central and western China. Additionally, mobile app–based interventions could be free from time and space constraints, greatly improving the efficiency and flexibility of interventions and reducing the cost, which can benefit more people. If this study proves that the 7-day digital HPV vaccine education intervention based on the IMB model can effectively improve the HPV vaccination rate, this convenient, low-cost, and easy-to-popularize intervention can be implemented among a wider range of people.

### Limitations

This study has several limitations. First, since HPV vaccines were approved to be used in China in 2016 and have not yet been covered by the National Immunization Program, the sample size cannot be accurately calculated due to the lack of the exact HPV vaccination rate or the HPV vaccine hesitancy rate in central and western China. Second, HPV vaccines need to be administered 2 or 3 times, but the follow-up period may not fully capture the entire vaccination process. As a result, it will be impossible to collect comprehensive data on the frequency and extent of vaccine hesitancy. Third, due to the nature of the intervention, participants in our study will not be blinded and contamination may occur. However, investigators and data analysts will all be blinded to the randomized allocation, and only the research assistants in each center will be aware of the allocation. Fourth, the research sites will be selected by convenience sampling, which would cause selection bias. Although this method is necessary, considering local cooperation and feasibility, it may limit the generalizability of our findings to other regions. Future studies could consider random sampling to enhance representativeness and reduce potential biases.

### Conclusion

To sum up, this will be the first study based on the IMB model for improving the HPV vaccination rate among 11-13-year-old girls in central and western China. This multicenter RCT can perhaps offer promising intervention actions for HPV vaccine hesitancy in the future. Future studies could focus on evaluating the effectiveness of HPV vaccination promotion materials across different geographical regions. Additionally, research could compare the impact of various intervention strategies to identify the most effective methods for increasing vaccine uptake.

## Appendix

Multimedia Appendix 1 SPIRIT (Standard Protocol Items: Recommendations for Interventional Trials) diagram for the schedule of enrollment, interventions, and assessments.

## Abbreviations

CNY: Chinese yuan
HPV: human papillomavirus
IMB: information-motivation-behavioral skills
RCT: randomized controlled trial
SPIRIT: Standard Protocol Items: Recommendations for Interventional Trials

## Data Availability

The data sets generated and analyzed during this study will be available from the corresponding author upon reasonable request.
