# Supplementary material for: The Effect of Interventions Based on the Information-Motivation-Behavioral Skills Model on the Human Papillomavirus Vaccination Rate Among 11-13-Year-Old Girls in Central and Western China: Protocol for a Randomized Controlled Trial
Source: JMIR Res Protoc. 2024 Nov 19;13:e58873. doi: 10.2196/58873 (PMC11615549; doi:10.2196/58873)
Supplement: Multimedia Appendix 1 [file resprot_v13i1e58873_app1.docx]

**Table S1.** SPIRIT^a^ diagram for the schedule of enrollment, interventions, and assessments.

| Schedule | | Enrollment timepoint (weeks) | Allocation timepoint (weeks) | Postallocation timepoint (weeks) | | | | | Close-out timepoint (weeks) |
| --- | --- | --- | --- | --- | --- | --- | --- | --- | --- |
|  | | –4 | 0 | +1 | +5 | +9 | +13 | +14 | etc. |
|  | |  |  |  |  |  |  |  |  |
| **Enrollment** | | | | | | | | | |
|  | Eligibility screen | X |  |  |  |  |  |  |  |
|  | Informed consent | X |  |  |  |  |  |  |  |
|  | 1:1 Randomization |  | X |  |  |  |  |  |  |
| **Interventions** | | | | | | | | | |
|  | IMB^b^-based Intervention for intervention group |  |  |  |  |  |  |  |  |
|  | Popular science education for control group |  |  |  |  |  |  |  |  |
|  | IMB-based Intervention for control group |  |  |  |  |  |  |  |  |
| **Assessments** | | | | | | | | | |
|  | Baseline questionnaire | X | X |  |  |  |  |  |  |
|  | Follow-up questionnaire |  |  |  | X | X | X |  |  |
|  | HPV^c^ vaccination rate |  |  | X | X | X | X | X | X |

^a^SPIRIT: Standard Protocol Items: Recommendations for Interventional Trials.

^b^IMB: information-motivation-behavioral skills.

^c^HPV: human papillomavirus.
